# Assessment of clinical workload for general and specialty genetic counsellors at an academic medical center: a tool for evaluating genetic counselling practices

**DOI:** 10.1038/npjgenmed.2016.10

**Published:** 2016-05-11

**Authors:** Brandie Heald, Lisa Rybicki, Diane Clements, Jessica Marquard, Jessica Mester, Ryan Noss, Monica Nardini, Jill Polk, Brittany Psensky, Christina Rigelsky, Allison Schreiber, Amy Shealy, Marissa Smith, Charis Eng

**Affiliations:** 1 Center for Personalized Genetic Healthcare, Genomic Medicine Institute, Cleveland Clinic, Cleveland, OH, USA; 2 Taussig Cancer Institute, Cleveland Clinic, Cleveland, OH, USA

## Abstract

With genomics influencing clinical decisions, genetics professionals are exponentially called upon as part of multidisciplinary care. Increasing demand for genetic counselling, a limited workforce, necessitates practices improve efficiency. We hypothesised that distinct differences in clinical workload exist between various disciplines of genetic counselling, complicating practice standardisation and patient volume expectations. We thus sought to objectively define and assess workload among various specialties of genetic counselling. Twelve genetic counsellors (GCs), representing 9.3 clinical FTE, in general or specialty (cancer, cardiovascular or prenatal) services at an academic health system developed a data collection tool for assessing time and complexity. Over a 6-week period, the data were recorded for 583 patient visits (136 general and 447 specialty) and analysed comparing general versus specialty GCs. Variables were compared with hierarchical linear models for ordinal or continuous data and hierarchical logistic models for binary data. General GCs completed more pre- and post-visit activities (*P*=0.011) and spent more time (*P*=0.009) per case. General GCs reported greater case discussion with other providers (*P*<0.001), literature review (*P*=0.026), exploring testing options (*P*=0.041), electronic medical record review (*P*=0.040), insurance preauthorization (*P*=0.05) and fielding patient inquiries (*P*=0.003). Lesser redundancy in referral indication was observed by general GCs. GCs in general practice carry a higher pre- and post-visit workload compared with GCs in specialty practices. General GCs may require lower patient volumes than specialty GCs to allow time for additional pre- and post-visit activities. Non-clinical activities should be transferred to support staff.

## Introduction

Genetic counselling is a process in which a healthcare provider assesses the likelihood of a disease having a genetic or hereditary basis from a patient’s personal and/or family health history; educates the patient about the genetic or hereditary disease(s) in question; explains the inheritance of the condition; discusses management options for the disease(s) and/or family planning options; when applicable, reviews the genetic testing process, obtains informed consent, facilitates ordering the genetic testing, and interpreting the genetic test results; and finally provides psychosocial support to the patient and his or her family.^1,2^ Healthcare providers who specialise in genetic counselling include medical geneticists, genetics nurses and genetic counsellors. Genetic counsellors are medical providers with a master’s degree in medical genetics or genetic counselling and are the focus of this study.

The early years of genetic counselling were primarily focused on reproductive counselling. However, there has been vast expansion of services into areas such as paediatrics, neurology, oncology, cardiology, metabolic disorders, complex adult onset disorders, newborn screening, infertility/ART/IVF, preconception and genomics, and even pharmacogenomics.^3^ In some sub-specialties of genetic counselling, such as oncology and preconception, the genetic counsellor might work autonomously. In other areas, such as paediatrics, the genetic counsellor might work alongside a medical geneticist. The geneticist has a critical role in conducting a physical examination, providing medical management recommendations and prescribing medications or treatments, as necessary.

The field of genetic counselling has shifted from utilisation of observed empiric risks (i.e., counselling about the risk of developing or passing on a disease based on the reported family history) to confirmatory diagnostic approaches with genetic testing. Commercial genetic tests are being released at a rapid pace, and are often updated in online directories, such as www.genetests.org, As of February 2016, there were over 4,000 genes and genetic disorders for which commercial genetic testing was available, resulting in over 55,500 tests (www.genetests.org). In addition to the growing number of genetic tests and increasing complexity of testing, including whole-exome sequencing and next-generation sequencing-based multi-gene panels, there is also greater awareness of genetic counselling services, which is leading to an increase in demand.^4^ In order to meet the demand for genetic counselling services, expansion of the work force and/or adjustment of current practice models is required to create greater access to genetic services. Adjustment of clinical practice models and establishing appropriate patient volume expectations for genetic counsellors should take into account that each discipline of genetic counselling serves different patient populations that may require varying levels of direct and indirect patient care. To that end, we sought to objectively assess the variation and care complexity or workload in clinical practice among general paediatric/adult, cancer, cardiovascular and prenatal genetic counselling services at a single large academic health system.

## Results

During the two 3-week periods of study (30 May 2014 to 1 July 2014 and 27 October 2014 to 12 November 2014), 583 patient visits (general (136), cancer (252), cardiovascular (103) and prenatal (92)) seen by 9.3 clinical full time equivalents (FTE) genetic counsellors were tracked. Per specialty, the numbers of patients seen per week per clinical FTE were as follows: general 7.3 total (5.1 new and 2.2 follow-up), cancer 14.7 total (13.8 new and 0.9 follow-up), cardiovascular 8.6 total (7.2 new and 1.4 follow-up) and prenatal 11.8 total (11.4 new and 0.4 follow-up).

On average, minimum time spent was greatest for general genetic counsellors compared with all specialties (*P*=0.004, Table 1). Visits in the general discipline were less likely to be genetic counsellor only appointments (*P*=0.013). Notably, 75.74% (103/136) of the general genetic counsellor visits were paired with a medical geneticist. The general genetic counsellor appointments were less likely to be new visits compared with the other specialties (*P*=0.010). A greater percentage of patients seen by the general group had a diagnosis/indication that was new to the genetic counsellor, though this did not reach statistical significance. The general group had greater anticipated and perceived complexity than the other disciplines (*P*=0.032 and 0.048, respectively). Compared with all other specialties, the general group was less likely to order testing for a known familial mutation (henceforth referred to as single-site genetic testing, *P*=0.041).

### Previsit

Among the previsit activities, the general genetic counsellors had a greater number of activities completed as well as time spent per patient (Table 2). Detailing the specific activities conducted previsit, general genetic counsellors more often reviewed the electronic medical record (*P*=0.040), discussed the case with other healthcare providers (*P*<0.001), conducted a literature review (*P*=0.026) and explored testing options (*P*=0.041). There was a trend that the general group conducted more insurance preauthorizations, but this did not reach statistical significance (*P*=0.06).

The indications for the genetic counselling referrals are summarised in Table 3 for each group. Greater redundancy of referral indications was observed by specialty genetic counsellors: 66% for cancer, 44% cardiovascular and 50% prenatal patients were referred for one of two most common indications, compared with only 16% of general patients.

### In-person visit

There were no differences observed in the total score or minimum time for the in-person visit (Table 4). Regarding the activities performed, there were differences observed that were appropriate per specialty. The general genetic counsellors more often obtained developmental history (*P*=0.002), asked questions for the Ohio Department of Health targeted to children ages ⩽5 years (*P*<0.001), obtained patient photographs (*P*=0.004), and spent more time waiting for the MD geneticist (*P*=0.004). These genetic counsellors less frequently collected reproductive history (*P*=0.012), collected information about prior evaluations or screening history (*P*=0.003), educated about genetic conditions (*P*=0.003), discussed inheritance (*P*=0.027), discussed management options (*P*=0.047), and consented for genetic testing (*P*=0.02).

### Post-visit

The general genetics had a higher number of post-visit activities completed and minimum time spent than all specialists combined (Table 5). The general genetic counsellors more often conducted insurance preauthorization (*P*=0.05), explored testing options (*P*=0.05), discussed the case with other providers (*P*=0.001) and handled patient inquiries (*P*=0.03).

## Discussion

With improvements in genetic testing technology and growing awareness among healthcare providers and patients, the demand for genetic counselling services is increasing.^4^ In order to meet this demand, there must be expansion of the number of providers in the field and/or accommodation of the current work force to higher patient volumes. Growth of the genetic counselling field is one of the 2015–2017 Strategic Initiatives being addressed by the National Society of Genetic Counsellors (NSGC, http://nsgc.org/p/cm/ld/fid=6). However, expanding patient volumes can potentially be addressed on an individual or institutional level.

This study found no differences for the in-person clinical session requirements for genetic counsellors providing general paediatric/adult, cancer, cardiovascular and prenatal services. However, general genetic counsellors required a significantly greater amount of time for pre- and post-visit activities. The general genetic counsellors also had greater variability among the referral indication and greater physician involvement than the other specialty practices. Therefore, it was not surprising to observe that the general genetic counsellors reported more often discussing the case with other providers, reviewing the literature, researching testing options and reviewing the electronic medical record.

Expansion of clinical services requires survey of the current patient care environment for each sub-specialty of the profession with mindful consideration of how to streamline patient care-related activities to maximise the genetic counsellor’s efficiency, in the context of the non-genetics clinical practices in a given institution. The tool used in this survey is capable of identifying patterns and differences in clinical genetic counselling practice. These patterns may reveal activities related to patient care that might be redundant or could be reallocated to support staff. For example, in this study, the general genetic counsellors conducted insurance preauthorization 7.4% and 19.1% of the time pre- and post-visit, respectively. On the basis of this, we identified institutional based financial analysts/counsellors to conduct the insurance preauthorizations on behalf of the genetic counsellors thus reducing the time burden for the genetic counsellor associated with this responsibility. In addition, given the repetitive nature of the cancer and cardiovascular genetic counsellor referrals, standardized templates for documentation were created prior to this study, which contributed to decreased time burdens in these sub-specialties.

Among the NSGC’s 2015–2017 Strategic Initiatives is to ‘define and promote best-practice models focused on high-quality, efficient delivery of genetic counselling services’ as well as ‘identify existing and needed tools and technology to support the efficiency of genetic counsellors.’ These are critical initiatives for genetic counsellors to address. Collectively, there are general similarities surrounding in-person-related patient care activities, which these data support. Divergence appears, however, on pre- and post-visit-related activities. This becomes particularly important as the field has entered an era driven by next-generation sequencing, which has brought whole-exome/-genome sequencing into routine clinical practice. At present, clinical whole-exome/-genome sequencing is not a routine test offered in cancer, cardiovascular or prenatal genetics settings. However, in paediatric and neurology genetics clinics, whole-exome/-genome sequencing has a critical role in identifying the genetic aetiology of those with intellectual disability or neurodevelopmental disorders, with a diagnostic yield of 25–40%.^5–11^ In addition to the potential diagnostic yield, whole-exome/-genome sequencing also results in incidental pathogenic mutations (which may or may not be clinically actionable), pharmacogenomic information, disease carrier status and a large number of variants of uncertain significance, all of which are manually reviewed by the ordering healthcare provider. A recent study by Williams *et al*. found that genetic counsellors were spending an average of 420 min (7 h) reviewing all available medical records in additional to time spent completing forms, making telephone calls, presenting to physicians/oversight committees and actually completing a genetic counselling and consenting session.^12^ Despite the fact that these healthcare providers were conducting standard genetic counselling activities, the scope of their activities varied greatly from genetic counselling sessions where testing for a single gene or syndrome was undertaken. Therefore, it is important to recognise these significant differences when creating practice models and establishing target patient volumes.

A small number of studies have evaluated the time-based efforts of genetics professionals.^13–17^ The original time study conducted in 1987 primarily evaluated the work of the medical geneticist in a paediatric setting, and found that clinical genetics services were labour-intensive.^13^ This was supported by a study in 2008 tracking the workflow of medical geneticists and genetic counsellors at a single institution, which found that each new patient required an average of 7 h of genetics professional time, with an average of 3.5 h for follow-up patient-related activities.^15^ In 2013, we evaluated the cancer genetic counsellors’ practice, and found that it was more efficient for genetic counsellors to see patients autonomously rather than paired with the medical geneticist.^17^ What all these studies lacked was a direct comparison among the sub-specialties of genetic counselling. Further, little is published about the workload of the genetic counsellor. As pointed out in the nursing literature, workload measures are not directly correlated with efficiency, complexity of the workload or the role of the work environment on these factors.^18–20^ Subjective measures of complexity also have a critical role in the perceived workload of the healthcare provider. Therefore, it is important to not only quantify the actual work done but to also capture the healthcare providers’ perceived burden of the work.

The major strength of our current study is the methodical approach of the data collection. Although the data obtained at this specific health system may not broadly be applicable to other genetic counselling practices, we believe the data collection tool is an adaptable instrument that could be used within any practice to identify opportunities to improve service delivery. A limitation of this study is the genetic counsellors’ years of experience could not be included in the analysis owing to the limited sample size of genetic counsellors. Experience certainly has a role in the efficiency of the provider and would be an important variable to assess in a larger study among genetic counsellors. This study was also limited by not evaluating time or activities related to results disclosure. This is an equally important task conducted by genetic counsellors, which is invariably associated with its own complexities and time requirements. For the purposes of this study, we used the type of test ordered as a surrogate marker for the associated clinical workload. For example, for the average patient, disclosure of a single-site genetic test result would be more straightforward than disclosure of exome sequencing results. An important area for future health services research would be to investigate the workload associated with results interpretation and disclosure for the various types of testing ordered by genetic counsellors.

Expanding patient volumes for genetic counsellors will help to create greater patient access to services. This can only occur when we have a greater understanding of what and how patient-related activities are being conducted, so opportunities to improve efficiencies can be identified. This will vary by sub-specialty of genetic counselling. Our data demonstrate that GCs in general practices may require lower patient volumes than specialty GCs to allow time for additional pre-/post-visit activities. We propose that determination of patient volume expectations for genetic counsellors should include consideration of specialty, variation in number of indications for referral and the genetic counsellor’s clinical FTE as well as what non-genetics tasks, e.g., insurance preauthorization, they are performing.

## Materials and methods

Over two 3-week periods (30 May 2014 to 1 July 2014 and 27 October 2014 to 12 November 2014), 12 genetic counsellors representing 9.3 clinical FTE, ranging from 1 to 28 years of experience, at a single academic institution prospectively tracked the data related to patient-care activity. The practice was housed within a genetics/genomics institute and divided into general paediatric/adult, cancer, cardiovascular and prenatal services. At this centre, the general paediatric/adult clinic serves patients with birth defects, developmental delays or other neurological issues, or any other indication not fitting within the other three specialty settings. Three of the genetic counsellors crossed disciplines. The following numbers of genetic counsellors were studied in each discipline: four general (3.1 clinical FTE), five cancer (2.85 clinical FTE), three cardiovascular (2.0 clinical FTE) and two prenatal (1.3 clinical FTE). Patients were seen as a combination of genetic counsellor only appointments and paired genetic counsellor and physician (geneticist) appointments. All initial consultations in the practice were conducted in person. Indications for follow-up visits include, but are not limited to: discussion of genetic test results, annual follow-up, updating the patient’s personal and/or family health history, review of new or additional genetic testing options.

The data collection tool was created by the genetic counsellors based on routine activities that are conducted surrounding patient care. Data were collected in REDCap. The genetic counsellors tracked 69 activities related to appointment preparation (16), in-person interactions (38) and post-appointment tasks (15; Supplementary Figure S1). Standardised definitions were used among the genetic counsellors to ensure consistency in scoring. In addition, the ranges (0–4, 5–15, 16–30, 31–60 or >60 min) of time spent for preparation, in-person interaction and follow-up were recorded.

For all cases, it was indicated whether this was a new diagnosis to the genetic counsellor, whether a trainee was involved with the case and what type of genetic testing was ordered (none, single-site, single gene/syndrome, small panel or non-invasive prenatal screening (NIPS), large panel or exome). Pre- and post-visit, the genetic counsellors subjectively self-rated the case as complex or simple.

Individual activity scores were summed to obtain complexity scores for previsit activities (potential range 0–32), in-person activities (0–77), post-visit activities (0–35) and all activities (0–144). The time intervals were utilised to calculate minimum time spent for the entire genetic counselling session by taking the minimum time for each interval (0, 5, 16, 31 or 60 min) and adding up those minimum times for each category (previsit, in-person and post-visit) of activity. Individual activities were analysed as having been done (score>0) or not done (score=0). Three activities were reported in <5 of 583 patient visits and were not analysed individually: family history by phone (previsit), write a letter of medical necessity (previsit) and complete school/employer forms (in person). Variables were reported using standard descriptive statistics.

Variables were compared by discipline with hierarchical linear models for ordinal or continuous data and hierarchical logistic models for the binary data to account for correlation within genetic counsellors, for varying number of patients seen by each genetic counsellor and for the different number of genetic counsellors within each discipline.

Data were described for all four disciplines and for specialties (cancer, cardiology and prenatal). Data were compared in two ways: once among the four disciplines and again for general versus specialty genetic counsellors. The comparisons among the four disciplines and of general versus specialty data are reported (Tables 2, 4 and 5). However, for the purposes of this manuscript, only the comparisons among general and specialty data are included in the Results and Discussion. Analyses were done with SAS Software, version 9.4 (SAS Institute, Inc, Cary, NC, USA). All statistical tests were two-sided, and *P* values ⩽0.05 were considered significant. No adjustments were made for multiple comparisons. The pretest hypothesis was that the general practice had greater complexity and thus higher time requirements, than the other three specialties.

## Figures and Tables

**Table 1:** Comparison among cancer, cardiovascular, general, prenatal and all specialists (cancer, cardiovascular and prenatal combined) groups for total score, minimum time spent, visit type, new or follow cases, new indication to the genetic counsellor, anticipated/perceived complexity and genetic tests ordered

*Variable*	*General (*n*=136)*	*Cancer (*n*=252)*	*Cardio (*n*=103)*	*Prenatal (*n*=92)*	*Specialties (*n*=447)*	P *value*
	*N* (%)	*N* (%)	*N* (%)	*N* (%)	*N* (%)	
*Total score (of 144 maximum; pre, in-person, and post)*						
Mean±s.d.	32±9	27±9	29±8	34±8	28±9	0.24
Median (range)	31 (13–63)	26 (7–58)	27 (14–72)	33 (16–69)	28 (7–72)	
						
*Minimum total time spent (min; pre, in-person and post)*						
Mean±s.d.	81±34	50±31	56±26	49±23	51±28	**0.004**
Median (range)	78 (15–180)	36 (10–180)	52 (5–180)	41 (21–136)	41 (5–180)	
						
*Visit type (GC only* versus *GC/MD)*						
GC only	33 (24.3)	234 (92.9)	47 (45.6)	91 (98.9)	372 (83.2)	**0.013**
						
*New or F/U*						
New	95 (69.9)	236 (93.7)	86 (83.5)	89 (96.7)	411 (91.9)	**0.010**
						
*New diagnosis to you*						
Yes	41 (30.1)	20 (7.9)	6 (5.8)	16 (17.4)	42 (9.4)	0.09
Anticipated case complexity (previsit)	(*n*=126)	(*n*=248)	(*n*=101)	(*n*=90)	(*n*=439)	
Complex	55 (43.7)	16 (6.5)	19 (18.8)	30 (33.3)	65 (14.8)	**0.032**
Perceived case complexity (post-visit)	(*n*=124)	(*n*=251)	(*n*=103)	(*n*=92)	(*n*=446)	
Complex	57 (46.0)	26 (10.4)	31 (30.1)	34 (37.0)	91 (20.4)	**0.048**
						
*Genetic testing ordered (each Yes* versus *No)*						
Single site	4(2.9)	27 (10.7)	11 (10.7)	8 (8.7)	46 (10.3)	**0.041**
Single gene/syndrome	28 (20.6)	63 (25.0)	9 (8.7)	13 (14.1)	85 (19.0)	0.62
Small panel or NIPS	34 (25.0)	89 (35.3)	21 (20.4)	62 (67.4)	172 (38.5)	0.25
Large panel or exome	11 (8.1)	0 (0.0)	12 (11.7)	1 (1.1)	13 (2.9)	0.12
						
GC1 (1 year exp)	34	9	0	0	9	
GC2 (2 years)	0	0	60	0	60	
GC3 (2 years)	13	67	0	0	67	
GC4 (5 years)	0	0	0	46	46	
GC5 (6 years)	0	76	0	0	76	
GC6 (8 years)	0	71	0	0	71	
GC7 (9 years)	0	15	0	0	15	
GC8 (9 years)	0	14	0	0	14	
GC9 (9 years)	0	0	29	0	29	
GC10 (11 years)	63	0	0	0	0	
GC11 (15 years)	26	0	0	46	46	
GC12 (28 years)	0	0	14	0	14	

Abbreviations: exp, experience; GC, Genetic counsellor; NIPS, non-invasive prenatal screening.

*P* value is the comparison of general versus specialists combined (i.e., comparison of column 1 and column 5). Statistically significant *P* values are shown in bold.

Data are also provided about the number of patients seen per GC with his or her years of experience listed.

**Table 2:** Previsit activities compared among general, cancer, cardiovascular, prenatal and all specialists combined (cancer, cardiovascular and prenatal)

*Variable*	*General (*n*=136)*	*Cancer (*n*=252)*	*Cardio (*n*=103)*	*Prenatal (*n*=92)*	*Specialties (*n*=447)*	P *value*^a^	P *value*^b^
	N *(%)*	N *(%)*	N *(%)*	N *(%)*	N *(%)*		
*Total previsit score (of 32 maximum)*							
Mean±s.d.	6±3	3±3	4±2	4±3	3±3	**0.035**	**0.002**
Median (range)	6 (0–14)	2 (0–15)	3 (0–10)	2 (0–14)	2 (0–15)		
							
*Time spent on previsit activities*							
0–4 min	3 (2.2)	128 (50.8)	22 (21.4)	28 (30.4)	178 (39.8)	**0.017**	**0.001**
5–15 min	21 (15.4)	74 (29.4)	34 (33.0)	35 (38.0)	143 (32.0)		
16–30 min	58 (42.6)	28 (11.1)	34 (33.0)	18 (19.6)	80 (17.9)		
31–60 min	38 (27.9)	10 (4.0)	11 (10.7)	11 (12.0)	32 (7.2)		
>60 min	16 (11.8)	12 (4.8)	2 (1.9)	0 (0.0)	14 (3.1)		
							
*Minimum time spent*							
Mean±s.d.	23±16	7±14	11±12	9±10	9±13		
Median (range)	16 (0–60)	0 (0–60)	5 (0–60)	5 (0–31)	5 (0–60)		
							
EPIC review	132 (97.1)	210 (83.3)	87 (84.5)	84 (91.3)	381 (85.2)	0.23	**0.04**
Request outside records	4 (2.9)	13 (5.2)	3 (2.9)	0	16 (3.6)	0.79 (s)	0.88
Review outside records	18 (13.2)	30 (11.9)	15 (14.6)	6 (6.5)	51 (11.4)	0.68	0.63
Discuss case with other providers	119 (87.5)	68 (27.0)	38 (36.9)	32 (34.8)	138 (30.9)	**0.003**	**<0.001**
Review pedigree	40 (29.4)	42 (16.7)	27 (26.2)	9 (9.8)	78 (17.4)	0.28	0.08
Prepopulate clinic note	78 (57.4)	140 (55.6)	49 (47.6)	85 (92.4)	274 (61.3)	0.48	0.44
Literature review	60 (44.1)	12 (4.8)	28 (27.2)	24 (26.1)	64 (14.3)	**0.023**	**0.026**
Run risk models	1 (0.7)	13 (5.2)	0	0	13 (2.9)	0.39 (s)	0.85
Explore testing options	57 (41.9)	14 (5.6)	35 (34.0)	15 (16.3)	64 (14.3)	**0.032**	**0.041**
Explore research options	6 (4.4)	14 (5.6)	3 (2.9)	0	17 (3.8)	0.32 (s)	0.78
Coordinate appointments	4 (2.9)	15 (6.0)	1 (1.0)	5 (5.4)	21 (4.7)	0.80	0.78
Create visual aids	7 (5.1)	12 (4.8)	0	3 (3.3)	15 (3.4)	0.81 (s)	0.25
Insurance preauthorization	10 (7.4)	2 (0.8)	2 (1.9)	0	4 (0.9)	0.16 (s)	0.06
Handle patient inquiries	15 (11.0)	41 (16.3)	9 (8.7)	7 (7.6)	57 (12.8)	0.51	0.85

(s) Could not be analysed as a binary outcome so a 0–1 score was analysed instead.

a Overall *P* value seeking differences among four groups (i.e., comparison among columns 1, 2, 3 and 4).

b *P* value comparing general with all specialists (i.e., comparison of column 1 and column 5). Statistically significant *P* values are shown in bold.

**Table 3:** Reasons for referrals by specialty

*Cancer*	N
P/FHx history breast cancer	121
P/FHx polyposis/ colorectal cancer	26
P/FHx *BRCA1/2* mutation	19
P/FHx ovarian cancer	14
P/FHx PTEN-hamartoma tumour syndrome	13
	
*P/FHx known syndrome*	13
ATM-cancer risk	
Familial adenatomous polyposis	
MUTYH-associated polyposis	
Juvenile polyposis syndrome	
Peutz–Jeghers syndrome	
Li–Fraumeni syndrome	
Hereditary paraganglioma	
P/FHx cancer	11
	
*Other*	11
Ashkenazi Jewish ancestry	
Barrett oesophagus	
Carcinoid	
Sebaceous carcinoma	
Kidney cancer	
Thyroid cancer	
Lung cancer	
Brain tumours	
Rule out Von–Hippel–Lindau	
	
P/FHx upper gastrointestinal cancer	8
P/FHx Lynch syndrome	6
P/FHx endometrial cancer	6
P/FHx pheocchromocytoma/paraganglioma	4
	
*Cardiovascular*	
P/FHx HCM	17
P/FHx connective tissue disease	15
	
*Other*	15
Blue sclera	
Bradycardia	
Stickler syndrome	
Diverticulosis	
Wolf–Parkinson–White	
Cardiac arrest	
Mitral valve prolapse	
Sudden cardiac death	
Cardiovascular disease	
*ACTA2*	
Loeys–Dietz	
Restrictive cardiomyopathy	
Multiple anomies with cardiomyopathy and dilated aorta	
	
P/FHx aortic aneurysm	13
P/FHx Marfan syndrome	11
P/FHx Ehlers–Danlos syndrome	9
P/FHx other aneurysm/dissection	9
P/FHx dilated cardiomyopathy	5
P/FHx long QT	3
P/FHx bicuspid aortic valve	3
P/FHx hereditary hemorrhagic telangiectasia	3
	
*General*	
Other	37
Cleidocranial dysplasia	
Rhabdomyolysis	
Rule out Alport syndrome	
Cerebral ventriculomegaly	
Cerebral cavernous malformations	
Right ventricular dilation, polyglandular autoimmune syndrome, neuropathy	
Kidney tumours, CPAM	
Speech delay and static encephalopathy	
Choroid plexus carcinoma	
Isolated lissencephaly sequence	
Skin tag of ear	
Common variable immune deficiency	
Hemihypertrophy X 2	
Nystagmus, variant in *FRMD7*	
Multiple medical complaints	
Fractures	
Auditory processing disorder	
Hemiplegic migraine	
Tetralogy of Fallot	
Failure to thrive	
Familial hypercholesterolaemia X 2	
Hirschsprung disease	
Ear anomaly, asymmetric cry	
MTHFR	
Bilateral amelia of upper limbs	
Fragile X testing	
Pseudohypoparathyroidism	
Macroglossia X 2	
Spina bifida	
Tricuspid valve atresia	
Hypoplastic left heart	
Abdominal pain, migraines, fatigue	
Abnormal amino acids, neuro symptoms	
Microcephaly, failure to thrive delays	
	
*P/FHx known syndrome*	22
CDG-1A	
Family history SBMA	
Usher syndrome	
Huntington disease X 3	
Brown-Vialetto-vanLaere syndrome X 2	
Osteogenesis imperfect	
Mowat-Wilson syndrome	
Beckwith–Wiedemann syndrome X 2	
Diamond–Blackfan anaemia	
	
DD/ID with or without other issues	13
	
*Chromosomal*	12
Down syndrome X 2	
Klinefelter’s syndrome	
12p deletion	
Turner syndrome	
22q11 deletion	
Abnormal microarray	
8;9 unbalanced translocation	
17q21.31deletion	
2q22.3q23.3 deletion	
Marker chromosome 15	
21q22.3 duplication	
	
NF evaluation	9
WES	8
Hearing loss	6
Epilepsy	6
Multiple congenital anomalies	5
Autism	5
Rule out porphyria	4
Ataxia	3
Mito	3
Cleft lip/palate	3
	
*Prenatal*	
Advanced maternal age	26
First trimester screening	20
	
*Family history of syndrome/birth defect/other health issue*	13
Hunter syndrome	
Thalassaemia	
Hydrocephaly	
*RET* mutation/Hirschsprung	
Asperger	
Duchene muscular dystrophy	
Intellectual disability	
Multiple congenital anomalies	
22q11.2 deletion syndrome	
Down syndrome, ID, fetal alcohol syndrome	
Hemophilia X 2	
Simpson–Golabi–Behmel syndrome	
	
*Fetal anomaly(ies)*	10
Heart defect X 3	
Bilateral cleft lip	
Ventriculomegaly	
Severe hydrocephaly	
Bilateral phocomelia, unilateral bowed femur	
Pericardial effusion	
Not otherwise specified X 2	
	
Fetal chromosomal abnormality	8
	
*Other*	8
Balanced translocation carrier	
Abnormal sequential screen	
Increased nuchal translucency	
Age related aneuploidy risk (non-AMA)	
Egg donor	
Possible thalassaemia	
Infertility due to partner Y microdeletions	
Abnormal Tay–Schas carrier results	
Advanced maternal age plus other issues	5
Multiple miscarriages	2

Abbreviations: HCM, hypertrophic cardiomyopathy; P/FHx, personal/family history.

**Table 4:** In-person visit activities compared among general, cancer, cardiovascular, prenatal, and all specialists combined (cancer, cardiovascular, and prenatal)

*Variable*	*General (*n*=136)*	*Cancer (*n*=252)*	*Cardio (*n*=103)*	*Prenatal (*n*=92)*	*Specialties (*n*=447)*	P *value*^a^	P *value*^b^
	N *(%)*	N *(%)*	N *(%)*	N *(%)*	N *(%)*		
*Total in-person score (of 77 maximum)*							
Mean±s.d.	20±6	20±6	21±6	25±6	21±6	0.64	0.75
Median (range)	20 (4–37)	20 (6–37)	21 (11–41)	25 (11–42)	22 (6–42)		
							
*Time spent on in-person activities*							
0–4 min	0 (0.0)	0 (0.0)	0 (0.0)	0 (0.0)	0 (0.0)	0.34	0.94
5–15 min	5 (3.7)	1 (0.4)	2 (1.9)	0 (0.0)	3 (0.7)		
16–30 min	30 (22.1)	43 (17.1)	37 (35.9)	30 (32.6)	110 (24.6)		
31–60 min	82 (60.3)	171 (67.9)	56 (54.4)	57 (62.0)	284 (63.5)		
>60 min	19 (14.0)	37 (14.7)	8 (7.8)	5 (5.4)	50 (11.2)		
							
*Minimum time spent*							
Mean±s.d.	31±14	33±13	27±12	28±10	30±12		
Median (range)	31 (5–60)	31 (5–60)	31 (5–60)	31 (16–60)	31 (5–60)		
							
Basic history collection	126 (92.6)	242 (96.0)	103 (100)	91 (98.9)	436 (97.5)	0.10 (s)	0.08
Social history collection	101 (74.3)	121 (48.0)	75 (72.8)	17 (18.5)	213 (47.7)	0.12	0.13
Reproductive history collection	3 (2.2)	171 (67.9)	5 (4.9)	86 (93.5)	262 (58.6)	**<0.001**	**0.012**
Pregnancy/birth history collection	66 (48.5)	3 (1.2)	48 (46.6)	42 (45.7)	93 (20.8)	**0.024**	0.09
Developmental history collection	89 (65.4)	5 (2.0)	15 (14.6)	6 (6.5)	26 (5.8)	**0.004**	**0.002**
Directed physical assessment	17 (12.5)	91 (36.1)	64 (62.1)	5 (5.4)	160 (35.8)	0.37	0.48
Assess Ohio Department of Health questions	46 (33.8)	1 (0.4)	5 (4.9)	0	6 (1.3)	**0.001 (s)**	**<0.001**
Collect evaluation/screening history	50 (36.8)	230 (91.3)	88 (85.4)	77 (83.7)	395 (88.4)	**0.040**	**0.003**
Review outside records	12 (8.8)	28 (11.1)	24 (23.3)	8 (8.7)	60 (13.4)	0.36	0.52
Collect family history	124 (91.2)	237 (94.0)	94 (91.3)	91 (98.9)	422 (94.4)	0.67	0.72
Create differential diagnosis	87 (64.0)	226 (89.7)	86 (83.5)	61 (66.3)	373 (83.4)	0.31	0.19
Run risk models for patient	0	78 (31.0)	0	1 (1.1)	79 (17.7)	**0.027 (s)**	0.19 (s)
Run risk models for family member(s)	0	13 (5.2)	0	1 (1.1)	14 (3.1)	0.27 (s)	0.31 (s)
Assess empiric risk for patient	36 (26.5)	67 (26.6)	89 (86.4)	48 (52.2)	204 (45.6)	**0.05**	0.22
Assess empiric risk for family member(s)	39 (28.7)	48 (19.0)	77 (74.8)	38 (41.3)	163 (36.5)	**0.05**	0.50
Discuss aneuploidy risk	1 (0.7)	0	1 (1.0)	71 (77.2)	72 (16.1)	**<0.001 (s)**	0.55
Discuss previous testing	73 (53.7)	82 (32.5)	24 (23.3)	53 (57.6)	159 (35.6)	**0.010**	0.06
Educate about genetic conditions	96 (70.6)	236 (93.7)	103 (100)	87 (94.6)	426 (95.3)	**0.013 (s)**	**0.003**
Educate about genetics	110 (80.9)	134 (53.2)	96 (93.2)	92 (100)	322 (72.0)	0.40 (s)	0.62
Discuss inheritance	75 (55.1)	223 (88.5)	87 (84.5)	87 (94.6)	397 (88.8)	0.12	**0.027**
Discuss recurrence risk	75 (55.1)	103 (40.9)	66 (64.1)	56 (60.9)	225 (50.3)	0.81	0.70
Review medical management options	37 (27.2)	218 (86.5)	64 (62.1)	20 (21.7)	302 (67.6)	**<0.001**	**0.047**
Review pregnancy management options	0	6 (2.4)	2 (1.9)	70 (76.1)	78 (17.4)	**<0.001 (s)**	0.30 (s)
Discuss genetic testing options	93 (68.4)	202 (80.2)	83 (80.6)	89 (96.7)	374 (83.7)	**0.038**	0.08
Explain genetic testing process	86 (63.2)	189 (75.0)	72 (69.9)	81 (88.0)	342 (76.5)	0.27	0.16
Review GINA	14 (10.3)	62 (24.6)	8 (7.8)	0	70 (15.7)	0.14 (s)	0.86
Discuss follow-up plan	121 (89.0)	212 (84.1)	75 (72.8)	85 (92.4)	372 (83.2)	0.13	0.30
Psychosocial assessment	49 (36.0)	143 (56.7)	27 (26.2)	44 (47.8)	214 (47.9)	0.87	0.71
Psychosocial counselling	43 (31.6)	116 (46.0)	32 (31.1)	65 (70.7)	213 (47.7)	0.58	0.46
Provide resources	23 (16.9)	78 (31.0)	23 (22.3)	17 (18.5)	118 (26.4)	0.57	0.30
Consent for genetic testing	41 (30.1)	171 (67.9)	38 (36.9)	62 (67.4)	271 (60.6)	**0.029**	**0.02**
Complete test requisition	18 (13.2)	123 (48.8)	12 (11.7)	33 (35.9)	168 (37.6)	0.36	0.35
Request outside records	10 (7.4)	13 (5.2)	8 (7.8)	0	21 (4.7)	0.30 (s)	0.39
Take patient photos	29 (21.3)	1 (0.4)	3 (2.9)	0	4 (0.9)	**0.05 (s)**	**0.004**
Language barrier/interpreter	8 (5.9)	3 (1.2)	5 (4.9)	6 (6.5)	14 (3.1)	0.19	0.39
Make referrals to specialists	23 (16.9)	19 (7.5)	14 (13.6)	14 (15.2)	47 (10.5)	0.32	0.14
Spend time waiting for the MD	63 (46.3)	17 (6.7)	7 (6.8)	2 (2.2)	26 (5.8)	**0.033**	**0.004**

Abbreviation: GINA, Genetic Information Non-Discrimination Act. (s) Could not be analysed as a binary outcome so a 0–1 score was analysed instead.

a Overall *P* value seeking differences among four groups (i.e., comparison among columns 1, 2, 3 and 4).

b *P* value comparing general with all specialists (i.e., comparison of column 1 and column 5). Statistically significant *P* values are shown in bold.

**Table 5:** Post-visit activities compared among general, cancer, cardiovascular, prenatal and all specialists combined (cancer, cardiovascular and prenatal)

*Variable*	*General (*n*=136)*	*Cancer (*n*=252)*	*Cardio (*n*=103)*	*Prenatal (*n*=92)*	*Specialties (*n*=447)*	P *value*^a^	P *value*^b^
	N *(%)*	N *(%)*	N *(%)*	N *(%)*	N *(%)*		
*Total post-visit score (of 35 maximum)*							
Mean±s.d.	6±2	3±2	4±3	4±2	4±2	0.08	**0.011**
Median (range)	6 (2–18)	3 (1–14)	4 (1–21)	4 (1–13)	3 (1–21)		
							
*Time spent on previsit activities*							
0–4 min	0	71 (28.2)	1 (1.0)	9 (9.8)	81 (18.1)	**0.045**	**0.009**
5–15 min	10 (7.4)	85 (33.7)	27 (26.2)	37 (40.2)	149 (33.3)		
16–30 min	52 (38.2)	72 (28.6)	52 (50.5)	32 (34.8)	156 (34.9)		
31–60 min	56 (41.2)	18 (7.1)	20 (19.4)	13 (14.1)	51 (11.4)		
>60 min	18 (13.2)	6 (2.4)	3 (2.9)	1 (1.1)	10 (2.2)		
							
*Minimum time spent*							
Mean±s.d.	27±15	10±12	17±12	13±11	12±12		
Median (range) (6)	31 (5–60)	5 (0–60)	16 (0–60)	10 (0–60)	5 (0–60)		
							
EPIC review	99 (72.8)	84 (33.3)	43 (41.7)	71 (77.2)	198 (44.3)	0.24	0.21
Complete clinic note	134 (98.5)	251 (99.6)	102 (99.0)	92 (100)	445 (99.6)	0.60 (s)	0.29
Write patient letter	1 (0.7)	6 (2.4)	0	0	6 (1.3)	0.47 (s)	0.90
Write letter of medical necessity	5 (3.7)	8 (3.2)	4 (3.9)	2 (2.2)	14 (3.1)	0.92	0.73
Package test kit	9 (6.6)	164 (65.1)	21 (20.4)	1 (1.1)	186 (41.6)	**<0.001**	0.09
Retrieve specimens	4 (2.9)	12 (4.8)	4 (3.9)	0	16 (3.6)	0.83 (s)	0.73
Request outside records	9 (6.6)	18 (7.1)	6 (5.8)	0	24 (5.4)	0.38 (s)	0.62
Review outside records	11 (8.1)	11 (4.4)	11 (10.7)	6 (6.5)	28 (6.3)	0.56	0.73
Insurance preauthorization	26 (19.1)	1 (0.4)	15 (14.6)	3 (3.3)	19 (4.3)	**0.020**	**0.05**
Literature review	18 (13.2)	6 (2.4)	8 (7.8)	12 (13.0)	26 (5.8)	0.10	0.24
Explore testing options	25 (18.4)	8 (3.2)	11 (10.7)	7 (7.6)	26 (5.8)	0.14	**0.05**
Explore research options	8 (5.9)	11 (4.4)	1 (1.0)	0	12 (2.7)	0.41 (s)	0.28
Coordinate appointments	13 (9.6)	11 (4.4)	7 (6.8)	10 (10.9)	28 (6.3)	0.47	0.32
Discuss case with other providers	116 (85.3)	37 (14.7)	38 (36.9)	49 (53.3)	124 (27.7)	**0.001**	**0.001**
Handle patient inquiries	38 (27.9)	21 (8.3)	18 (17.5)	8 (8.7)	47 (10.5)	0.13	**0.03**

(s) Could not be analysed as a binary outcome so a 0–1 score was analysed instead.

a Overall *P* value seeking differences among four groups (i.e., comparison among columns 1, 2, 3 and 4).

b *P* value comparing general with all specialists (i.e., comparison of column 1 and column 5). Statistically significant *P* values are shown in bold.
