# Supplementary material for: Assessment of clinical workload for general and specialty genetic counsellors at an academic medical center: a tool for evaluating genetic counselling practices
Source: NPJ Genom Med. 2016 May 11;1:16010–. doi: 10.1038/npjgenmed.2016.10 (PMC5685297; doi:10.1038/npjgenmed.2016.10)
Supplement: Supplementary Figure S1 [file npjgenmed201610-s1.doc]

**Supplemental Figure 1.** Data collection tool and standardized definitions utilized by the genetic counselors

MRN: ______________________________________

DOV:_____________________________________

GC Last Name: ________________________________

Visit type: □ GC Only □ GC/MD

New or F/U: □ New □ F/U for est dx □ F/U new indication □ F/U results

□ Other: ___________

Specialty: □ Cancer □ Cardiovascular □ General □ Prenatal □ Other: ___________

Trainee involved: □ Yes □ No

Reason for Referral: _____________________________

New diagnosis to you: □ Yes □ No

Anticipated case complexity (pre-case): □ Simple □ Complex □ Unknown

Genetic testing ordered (Check all that apply):

□ None

□ Single site

□ Single gene/syndrome

□ Small panel

□ Large panel or exome

**Perceived case complexity (post-case): □ Simple □ Complex □ Unknown**

| **PRE-APPOINTMENT** |  | Pregnancy management options | **0 1 2 3** |
| --- | --- | --- | --- |
| EPIC review | **0 1 2 3** | Genetic testing options | **0 1 2 3** |
| Outside records requested | **0 1 2** | Genetic test process | **0 1 2 3** |
| Outside records review | **0 1 2 3** | Protections against discrimination | **0 1** |
| Case discussion w/ other provider(s) | **0 1 2** | Discuss follow up plan | **0 1** |
| Pedigree by phone | **0 2** | Psychosocial assessment patient or family | **0 1 2 3** |
| Review pedigree previously obtained | **0 1** | Psychosocial counseling patient or family | **0 1 2 3** |
| Prepopulate note | **0 1** | Provide resources | **0 1 2 3** |
| Literature review | **0 1 2 3** | Obtain consent for genetic testing | **0 1** |
| Risk models | **0 1** | Complete test requisition | **0 1** |
| Explore testing options | **0 1 2** | Non CCHS medical records requests | **0 1 2** |
| Explore research options | **0 1 2** | Patient photos | **0 1** |
| Coordinate appointments | **0 1 2** | Language barrier | **0 1** |
| Create visual aids | **0 1 2** | Completing school/employer forms | **0 1** |
| Insurance preauthorization | **0 2** | Facilitate referrals to specialists | **0 1 2 3** |
| Write LMN | **0 2** | Spent time waiting for MD | **0 1** |
| Patient inquiries | **0 1 2** | Range of time spent | **1 2 3 4 5** |
| Range of time spent | **1 2 3 4 5** | **POST-APPOINTMENT** |  |
| **APPOINTMENT** |  | Epic review | **0 1 2 3** |
| No-show/late cancellation | **Y N** | Complete clinic note | **0 1 2 3** |
| Basic history collection | **0 1 2 3** | Patient letter | **0 1 2 3** |
| Social history collection | **0 1** | Write LMN | **0 1 2 3** |
| Reproductive history collection | **0 1** | Complete TRF | **0 1** |
| Pregnancy/birth history collection | **0 1** | Package test kit | **0 1** |
| Developmental history collection | **0 1** | Coordinate specimen retrieval | **0 1 2** |
| Directed assessment | **0 1** | Request outside records | **0 1 2** |
| ODH questions | **0 1** | Review outside records | **0 1 2 3** |
| Evaluations/screening history collection | **0 1** | Insurance preauthorization | **0 1 2** |
| Review outside records | **0 1 2 3** | Literature review | **0 1 2 3** |
| Family history | **0 1 2 3** | Explore testing options | **0 1 2** |
| Create differential diagnosis | **0 1 2 3** | Explore research options | **0 1 2** |
| Assessment of patient’s risk (models) | **0 2** | Coordinate appointments | **0 1 2** |
| Assessment of family member’s(s’) risk(s) (models) | **0 2** | Case follow up with other providers | **0 1 2** |
| Assessment of patient’s risk | **0 1 2 3** | Patient inquiries | **0 1 2** |
| Assessment of family member’s(s’) risk(s) | **0 1 2 3** | Range of time spent | **1 2 3 4 5** |
| Aneuploidy risk | **0 1 2 3** |  |  |
| Discuss previous testing | **0 1 2 3** |  |  |
| Educate about genetic condition(s) | **0 1 2 3** |  |  |
| Educate about genetics | **0 1 2 3** |  |  |
| Discuss mode of inheritance | **0 1** |  |  |
| Discuss recurrence risk | **0 1 2 3** |  |  |
| Medical management options | **0 1 2 3** |  |  |

**Completing Complexity Genetic Counseling Score Card**

**If appropriate, quantities should be indicated.**

Documentation in the chart should always support the selections on the score sheet. Below are the descriptions of each intervention/item and when they should be used.

**PRE-APPOINTMENT**

**EPIC review**

0-no records in EPIC for review

1 – review of limited records (1-2 clinic notes or imaging/pathology reports)

2 – review of a moderate amount of records (several similar reports, such as 5+ colonoscopy and pathology reports or assessments and clinical notes from >2 providers)

3 – review of a significant amount of records (numerous clinical notes, imaging reports, and other records reviewed in detail)

**Outside records requested**

0-no records requested

1-release obtained and sent to 1 outside facility

2-release obtained and sent to >2 outside facilities

**Outside records review**

0-no records available for review

1 – review of limited records (1-2 clinic notes or imaging/pathology reports)

2 – review of a moderate amount of records (several similar reports, such as 5+ colonoscopy and pathology reports or assessments and clinical notes from >2 providers)

3 – review of a significant amount of records (numerous clinical notes, imaging reports, and other records reviewed in detail)

**Family history by phone (including adopted patient**)

0 – not done

1 – to be used for patients who are adopted with no information about biological family. History of any offspring should be documented

2 – to be used when there is a previously obtained pedigree from a healthcare provider or the patient has completed Cleveland Clinic’s MyFamily. In this case the genetic counselor is confirming previously obtained information and updating new information. Adopted patients with information about the biological family can fit into this category

3 – the genetic counselor obtains a 3-4 generation pedigree. Adopted patients with information about the biological family can fit into this category

**Review pedigree previously obtained**

0-no pedigree available for review

1-pedigree reviewed

**Prepopulate note**

0-no pre-population performed

1-visit encounter started before patient’s arrival

**Literature review**

0-no literature review performed/necessary

1-brief review (1 review article or clinical guidelines)

2-moderate review (>1 review articles, primary literature, clinical guidelines)

3- extensive review (in-depth review of primary literature, review articles, clinical guidelines; researching literature to identify previous reports of identified VUS or other test result fits here)

**Create differential diagnosis** – listing of condition(s), syndrome(s), and/or exposures that may be the explanation for the patient’s personals and/or family history.

0 – not done

1 – differential includes 1 syndrome/condition

2 – differential includes 2-3 syndromes/conditions

3 – differential includes >3 syndromes/conditions

**Assessment of patient’s risk (models)** – evaluation of patient’s risk (of cancer and/or mutational status) based on personal/family history using validated models, such as BRCAPro, Tyrer-Cusak, Penn Model, MMR Pro, PTEN risk assessment model.

0 – not done

2 – done

**Assessment of family member’s(s’) risk(s) (models)** - evaluation of patient’s relatives risk (of cancer and/or mutational status) based on personal/family history using validated models, such as BRCAPro, Tyrer-Cusak, Penn Model, MMR Pro, PTEN risk assessment model.

0 – not done

2 – done

**Assessment of patient’s empiric risk** – estimate of patient’s disease risk based on reported history and peer-reviewed literature.

0 – not done

1 – you know the number by heart or obtain it from a table you frequently reference

2 – you had to complete a brief literature review to obtain the information

3 – you had to complete a detailed literature review to obtain the information

**Assessment of family member’s(s’) empiric risk(s) –** estimate of patient’s relatives’ disease risk based on reported history and peer-reviewed literature.

0 – not done

1 – you know the number by heart or obtain it from a table you frequently reference

2 – you had to complete a brief literature review to obtain the information

3 – you had to complete a detailed literature review to obtain the information

**Aneuploidy risk** – identify age related aneuploidy risk based on peer-reviewed literature.

0 – not done

1 – you know the number by heart or obtain it from a table you frequently reference

2 – you had to complete a brief literature review to obtain the information

3 – you had to complete a detailed literature review to obtain the information

**Explore testing options**

0-no testing options explored

1-brief review of available testing options at pertinent clinical labs

2-in-depth review of testing options at clinical labs (includes looking up pricing information, authorization requirements)

**Explore research options**

0-not performed

1-brief review of GeneTests for availability of research options; prepare paperwork for CCF research study option(s)

2-in-depth review of research options from GeneTests or primary literature, to include contacting researcher to investigate eligibility requirements and enrollment processes

**Coordinate appointments**

0-no other appointments coordinated

1-1 other appointment coordinated

2->2 other appointments coordinated

**Create visual aids**

0-not done

1-existing visual aids updated

2-new visual aids created

**Insurance preauthorization**

0-not done

2-contact with patient’s insurance company for pre-authorization work

**Write LMN**

0 – not done

1 – solely used template, this also includes copying previous patient letters as a template

2 – used template with minor additions or edits, this also includes copying previous patient letters as a template

3 – no template, written completely from scratch

**Patient Inquires**

0-no pre-visit communication with patient

1-brief conversation or short email communication to determine if other genetic testing previously performed on patient/family, requested to bring information to visit or send in ahead of time

2-extensive conversation/emailing with patient/family regarding reason for visit, what happens at appointment, possibility of testing performed

**Case discussion with other providers**

0 – not done

1 – brief conversation with fellow genetic counselors, MDs, or other healthcare providers

2 – extensive conversation and/or presentation at multidisciplinary care meeting

**Time**

1 – 0-5 minutes

2 – 5-15 minutes

3 – 16-30 minutes

4 – 31-60 minutes

5 – 60+ minutes

**APPOINTMENT (MUST BE DONE FACE TO FACE)**

**Basic history collection**

0 – not done

1 – collection only of history of present illness

2 – collection of history of present illness (<3 body systems involved), past medical history, and past surgical history

3 – collection of history of present illness (≥3 body systems involved), past medical history, and past surgical history

**Social history collection**

0 – not done

1 – questions about social history such as who lives at home, occupation, current school year, exposure history, smoking history, drug history, and alcohol history

**Reproductive history collection (female only)**

0 – not done

1 – collection of history related to reproduction could include age at first menses, age at menopause, number of pregnancies/live births/abortions (spontaneous and therapeutic), infertility history, and hormone use

**Pregnancy/birth history collection**

0 – not done

1 – collection of history related to that patient’s gestation and birth

**Developmental history collection**

0 – not done

1 – collection of history related to physical and mental development including, but not limited, age at developmental milestones, age-appropriate academic achievements

**Directed assessment**

0-not done

1-brief discussion/evaluation of physical characteristics such as head size measurement, cutaneous features for further physician exam

**ODH questions (children under 5 only)**

0 – not done

1 – collection of information about utilization of state-funded services

**Evaluation/screening history collection**

0 – not done

1 – collection of imaging and/or screening undertaken including frequency and/or date(s) of recent exams and findings relevant to subspecialty

**Review outside records** – review of outside CCHS medical records for the patient and/or family that are submitted prior to or at the time of the appointment and reviewed in real time with the patient

0 – not done

1 – review of limited records (examples include 1-2 ultrasound reports or colonoscopy and pathology reports)

2 – review of a moderate amount of records (examples include several similar reports, such as 5+ colonoscopy and pathology reports or assessments and clinical notes from >2 providers)

3 – review of a significant amount of records (examples include assessments from numerous providers, healthcare systems, or years of evaluations)

**Family history (including adopted patient**)

0 – not done

1 – to be used for patients who are adopted with no information about biological family. History of any offspring should be documented

2 – to be used when there is a previously obtained pedigree from a healthcare provider or the patient has completed Cleveland Clinic’s MyFamily. In this case the genetic counselor is confirming previously obtained information and updating new information. Adopted patients with information about the biological family can fit into this category

3 – the genetic counselor obtains a 3-4 generation pedigree. Adopted patients with information about the biological family can fit into this category

**Create differential diagnosis** – listing of condition(s), syndrome(s), and/or exposures that may be the explanation for the patient’s personals and/or family history.

0 – not done

1 – differential includes 1 syndrome/condition

2 – differential includes 2-3 syndromes/conditions

3 – differential includes >3 syndromes/conditions

**Assessment of patient’s risk (models)** – evaluation of patient’s risk (of cancer and/or mutational status) based on personal/family history using validated models, such as BRCAPro, Tyrer-Cusak, Penn Model, MMR Pro, PTEN risk assessment model.

0 – not done

2 – done

**Assessment of family member’s(s’) risk(s) (models)** - evaluation of patient’s relatives risk (of cancer and/or mutational status) based on personal/family history using validated models, such as BRCAPro, Tyrer-Cusak, Penn Model, MMR Pro, PTEN risk assessment model.

0 – not done

2 – done

**Assessment of patient’s empiric risk** – estimate of patient’s disease risk based on reported history and peer-reviewed literature.

0 – not done

1 – you know the number by heart or obtain it from a table you frequently reference

2 – you had to complete a brief literature review to obtain the information

3 – you had to complete a detailed literature review to obtain the information

**Assessment of family member’s(s’) empiric risk(s) –** estimate of patient’s relatives’ disease risk based on reported history and peer-reviewed literature.

0 – not done

1 – you know the number by heart or obtain it from a table you frequently reference

2 – you had to complete a brief literature review to obtain the information

3 – you had to complete a detailed literature review to obtain the information

**Aneuploidy risk** – identify age related aneuploidy risk based on peer-reviewed literature.

0 – not done

1 – you know the number by heart or obtain it from a table you frequently reference

2 – you had to complete a brief literature review to obtain the information

3 – you had to complete a detailed literature review to obtain the information

**Discuss previous testing**

0 – not done

1 – review the results of single gene/syndrome and implications of genetic testing previously performed for the patient and/or their relative

2 – review the results of smaller panel and implications of genetic testing previously performed for the patient and/or their relative

3 – review the results of larger panel or exome/genome and implications of genetic testing previously performed for the patient and/or their relative.

**Educate about genetic condition(s)** – review the natural history of the suspected or confirmed genetic condition(s)

0 – not done

1 – 1 syndrome

2 – 2 syndromes

3 – ≥3 syndromes

**Educate about genetics** – discuss genes, chromosomes, mutations, etc

0 – not done

1 – broad level overview of genes and/or chromosomes

2 – slightly greater details of genes and/or chromosomes

3 – detailed discussion of gene function/role, pathways, or chromosomal replication and/or errors

**Discuss mode of inheritance** – review the mode of inheritance and discuss the risk or potential risks to other relatives

0 – not done

1 – done

**Discuss recurrence risk** – explain the risk of disease to other family members when a genetic basis is not identified.

0 – not done

1 – recurrence risk was discussed in a limited setting (example includes risk to a future pregnancy)

2 – recurrence risk was reviewed for all first degree relatives

3 – recurrence risk was reviewed for more than first degree relatives and/or there are many pathways or factors contributing to the recurrence risks that were reviewed (example includes discussing risks if patient was tested positive/negative vs if another family members was tested positive/negative)

**Review medical management options** – discuss the management options or professional/expert recommendations for the suspected or confirmed genetic condition(s)

0 – not done

1 – you reviewed a guideline that you know by heart or is readily available

2 – you completed a basic literature review to obtain this information and review it with the patient

3 – you completed a detailed literature review and/or extensive discussion with ‘experts’ in the field to obtain said guidelines and review it with the patient

**Review pregnancy management options** – discuss options of developing birth plan, continuing pregnancy, termination, and/or adoption

0 – not done

1 – information was presented with no further discussion

2 – information was presented with limited discussion/questions

3 – information was presented and extensive assistance with plan development or decision making was required

**Discuss genetic testing options –** this includes a discussion of review potential test results including positive, negative, uncertain, and unanticipated (as appropriate)

0 – not done

1 – review single gene/syndrome testing and implications of genetic testing

2 – review smaller panel or microarray and implications of genetic testing. Single gene testing may be included in this category if multiple reflexive tests are being ordered.

3 – review the results of larger panel or exome/genome and implications of genetic testing

**Explain genetic testing process** – review type of sample, turn-around-time, and insurance reimbursement/prior authorization

0 – not done

1 – done

**Review protections against discrimination** – review the Genetic Information Non-Discrimination Act

0 – not done

1 – done

**Discuss follow up plan** – make arrangements for results disclosure or medical genetics follow up

0 – not done

1 – done

**Psychosocial assessment patient or family –** identify psychosocial issues impacting the patient or family at the time of encounter

0 – not done

1 – superficial assessment not discussed with patient but must be mentioned in the note

2 – assessment of 1-2 issues discussed with the patient and must be documented in the note

3 – detailed assessment of patient discussed with the patient and must be documented in the note

**Psychosocial counseling patient or family –** provide basic counseling for psychosocial issues that arise at the time of the encounter

0 – not done

1 – minimal counseling in the way of empathy statements

2 – 1-2 issues that were discussed during the session but were a natural part of the conversation

3 – extensive counseling and may or may not have impeded the medical issue at hand

**Provide resources –** provide the patient and/or family with supportive resources such as educational materials or support group information

0 – not done

1 – done with resources that are readily at your finger tips

2 – done with resources that required a quick review of the literature or the internet

3 – done with resources that required significant research

**Obtain consent for genetic testing** – obtain written consent

0 – not done

1 – done

**Complete test requisition –** complete laboratory specific requisition forms

0 – not done

1 – done

**Non CCHS medical record requests** – obtain consent from patient or parent/guardian to get outside records

0 – not done

1 – records requested from only 1 institution

2 – records requested from >1 institution

**Patient photos** – upon obtaining consent, take medical photos of the patient

0 – not done

1 – done

**Language barrier** – use of interpreter to provide services

0 – not done

1 - done

**Completing school/employer forms** – complete the forms at the patient’s request

0 – not done

1 – done

**Facilitate referrals to specialists** – provide referrals to specialists related to patient’s/family’s condition. This may include placing EPIC orders.

0 – not done

1 – done but with routine providers that are our collaborators and required limited thought

2 – you had to figure out who they needed to see for a limited number of providers

3 – you had to refer to numerous specialists which required significant research or time to figure out.

**Time**

1 – 0-5 minutes

2 – 5-15 minutes

3 – 16-30 minutes

4 – 31-60 minutes

5 – 60+ minutes

**POST-APPOINTMENT**

**EPIC review**

0-no records in EPIC for review

1 – review of limited records (1-2 clinic notes or imaging/pathology reports)

2 – review of a moderate amount of records (several similar reports, such as 5+ colonoscopy and pathology reports or assessments and clinical notes from >2 providers)

3 – review of a significant amount of records (numerous clinical notes, imaging reports, and other records reviewed in detail)

**Complete clinic note**

0 – not done

1 – solely used template

2 – used template with minor additions or edits

3 – no template, assessment and recommendations completely from scratch or partially used template with major additions and edits

**Patient letter**

0 – not done

1 – solely used template, this also includes copying previous patient letters as a template

2 – used template with minor additions or edits, this also includes copying previous patient letters as a template

3 – no template, written completely from scratch

**Write LMN**

0 – not done

1 – solely used template, this also includes copying previous patient letters as a template

2 – used template with minor additions or edits, this also includes copying previous patient letters as a template

3 – no template, written completely from scratch

**Complete TRF**

0 – not done

1 – done

**Package test kit**

0 – not done

1 – done

**Coordinate specimen retrieval**

0 – not done (by you, does not count if someone else in the department got it)

1 – you had to work to find someone to get it for you

2 – you are running around getting patient samples

**Request outside records**

0 – not done

1 – done, from 1 outside facility

2 – done, from 2 or more outside facilities

**Review outside records**

0 – not done

1 – review of limited records (examples include 1-2 ultrasound reports or colonoscopy and pathology reports)

2 – review of a moderate amount of records (examples include several similar reports, such as 5+ colonoscopy and pathology reports or assessments and clinical notes from >2 providers)

3 – review of a significant amount of records (examples include assessments from numerous providers, healthcare systems, or years of evaluations)

**Insurance preauthorization**

0 – not done

1 – requires only one call that was completed in a timely manner

2 – requires multiple calls or calls that are lengthier than anticipated for insurance companies

**Literature review**

0 – not done

1 – a quick google or pubmed search solved it

2 – you reviewed a limited number of papers or only read abstract

3 – detailed review of papers and abstracts

**Explore testing options**

0-no testing options explored

1-brief review of available testing options at pertinent clinical labs

2-in-depth review of testing options at clinical labs (includes looking up pricing information, authorization requirements)

**Explore research options**

0-not performed

1-brief review of GeneTests for availability of research options; prepare paperwork for CCF research study option(s)

2-in-depth review of research options from GeneTests or primary literature, to include contacting researcher to investigate eligibility requirements and enrollment processes

**Coordinate appointments**

0-no other appointments coordinated

1-1 other appointment coordinated

2->2 other appointments coordinated

**Case discussion with other providers**

0 – not done

1 – brief conversation with fellow genetic counselors, MDs, or other healthcare providers

2 – extensive conversation and/or presentation at multidisciplinary care meeting

**Patient inquiries**

0 – not done

1 – field an email or phone call

2 – field numerous communications and/or lengthy communications

**Time**

1 – 0-5 minutes

2 – 5-15 minutes

3 – 16-30 minutes

4 – 31-60 minutes

5 – 60+ minutes
